# Standardized Templates for Thoracic Anesthesia Practitioners: A Quality Improvement Needs Assessment to Support Onboarding and Protocol Adherence

**DOI:** 10.7759/cureus.112204

**Published:** 2026-07-07

**Authors:** Taha B Aljishi

**Affiliations:** 1 Anesthesiology, King Fahad Specialist Hospital, Dammam, SAU; 2 Anesthesiology and Pain Medicine, The Ottawa Hospital, Ottawa, CAN

**Keywords:** anesthesia, checklist, education, fellow, guideline, lung, medical, quality, resident, thoracic

## Abstract

Background

Onboarding in anesthesiology varies widely across institutions and often emphasizes logistics over clinical workflow integration, contributing to cognitive load and inconsistent early practice. These challenges may be amplified in thoracic anesthesia, where complex perioperative management requires specialized skills and coordinated workflows. Although structured perioperative pathways, such as Enhanced Recovery After Surgery (ERAS) protocols, improve outcomes when consistently implemented, adherence depends on reliable, accessible clinical guidance. This quality improvement needs assessment evaluated thoracic anesthesia practitioners' perceptions of standardized, workflow-integrated templates to support onboarding and protocol adherence.

Methods

A pre-implementation needs assessment was conducted among anesthesia residents, fellows, and faculty to evaluate the perceived usefulness of standardized template systems for thoracic cases, with a primary focus on the ERAS protocol. The survey assessed anticipated effects on confidence and clinical focus (e.g., shifting effort from learning local norms to patient optimization and patient-centered care) and identified perceived barriers and concerns to implementation.

Results

Most respondents were staff anesthesiologists. Exposure to thoracic anesthesia varied. Most participants reported prior experience with ERAS protocols. Participants perceived that access to a standardized thoracic ERAS protocol would increase confidence in managing cases and facilitate more rapid integration into new clinical environments. Respondents also reported that standardized protocols would support greater focus on patient optimization rather than determining local standards of care. Reported concerns included potential reductions in clinical flexibility and the need for regular protocol updates, while integration within the electronic health record was identified as the preferred mode of access.

Conclusion

Respondents perceived standardized thoracic ERAS protocols as useful for improving confidence, integration, and clinical focus, with EHR integration preferred. Concerns centered on flexibility and protocol updates. Findings support implementation feasibility and inform a post-implementation evaluation.

## Introduction

Onboarding anesthesia practitioners presents ongoing challenges in large academic hospitals. Variability in institutional protocols and available resources can increase cognitive load and contribute to inconsistent practice during early integration [1]. These challenges may be amplified in thoracic anesthesia, where complex airway management, lung isolation, and perioperative planning for high-risk lung resections require specialized skills and coordinated workflows. In perioperative care, reliable adherence to structured pathways, such as Enhanced Recovery After Surgery (ERAS) protocols and institutional clinical guidelines, is important for optimizing outcomes and resource use, including after lung resection surgery [2,3].

Many onboarding processes emphasize logistical tasks while providing limited guidance on professional expectations and local clinical workflows. This gap may increase safety risks and contribute to clinician stress and burnout [4-6]. In anesthesiology, the transition into training and independent practice requires rapid development of technical and decision-making skills. It may be particularly challenging when expectations and case-discussion formats vary across supervisors [1]. Orientation practices also vary substantially across programs in content, timing, and delivery, highlighting the absence of a standardized onboarding framework [7,8]. Evidence from other specialties supports the value of structured onboarding, and broader consolidation into larger hospital systems further underscores the need for robust onboarding and mentorship to support consistent quality of care [4,5].

Technology-enabled onboarding strategies, including structured electronic health record (EHR) training and mobile or digital resources, have been used to improve preparedness and access to institutional information [9-11]. Building on these approaches, standardized thoracic anesthesia templates (e.g., a thoracic ERAS protocol and related perioperative guidance) may offer a practical, low-burden method to support onboarding and standardize practice.

This quality improvement needs assessment evaluates thoracic anesthesia practitioners’ perceptions of distributing or embedding thoracic anesthesia-specific standardized templates within clinical workflows (including potential EHR integration), and identifies anticipated benefits, barriers, concerns, and preferred access methods to inform subsequent implementation and evaluation.

## Materials and methods

Subjects

The subjects of interest were anesthesia practitioners involved in the delivery of anesthesia for thoracic surgery cases, including residents, fellows, and senior or newly hired staff, who were currently pursuing training or practice in Canadian or international hospitals.

Measures

Key outcomes included perceived effects on confidence, onboarding/integration, ability to focus on patient optimization (rather than learning local norms), and reassurance to thoracic surgeons, as well as perceived concerns, barriers, and preferred access methods; these were captured using 5-point Likert-type response options anchored from “not at all” to “very much” (or “strongly disagree” to “strongly agree”), along with single- and multiple-selection items.

Data collection

This is a needs-assessment-based quality improvement project targeting anesthesia practitioners involved in thoracic surgery. The assessment tool was developed following a comprehensive literature review using PubMed and Google Scholar, supplemented by expert input and pilot testing to test clarity, response rates, and early trends. The pilot testing was conducted within the thoracic anesthesia subspecialty group in our department. Based on iterative feedback, the survey underwent multiple rounds of refinement to improve clarity and better align the questions with the intended objectives. In particular, the questionnaire was revised to focus specifically on thoracic anesthesia practice and to evaluate perceptions of a defined protocol--ERAS--rather than referring more broadly to standardized templates or institutional protocols. The assessment focused on templates related to Enhanced Recovery After Thoracic Surgery (ERATS) protocols. It consisted of three sections: (1) sociodemographic questions, (2) perceptions of standardized templates, and (3) barriers and concerns. Question formats include multiple-choice, single-response, and Likert scale items (Appendices).

Recruitment was conducted via a department-wide email distributed by the departmental secretary to all targeted anesthesia practitioners across all Ottawa Hospital campuses. The invitation email was sent once, with a single reminder, and data were collected over a one-month period. The number of eligible recipients could not be precisely determined; therefore, a response rate was not calculated. All surveys were completed in full, and no missing items were observed.

Inclusion and exclusion

Anesthesia residents, fellows, and both senior and newly hired anesthesia staff engaged in thoracic anesthesia clinical practice within Canadian or international hospitals, including those with less than one year of practice, were included in the study. Rotating physicians from other specialties, anesthesia interns, medical students, allied health personnel, and attending anesthesiologists not directly targeted by the onboarding process were excluded (Table 1).

**Table 1:** Inclusion and exclusion criteria for study participants

Inclusion Criteria	Exclusion Criteria
Anesthesia residents	Rotating physicians from other specialties
Anesthesia fellows	Anesthesia interns
Senior anesthesia staff	Medical students
Newly hired anesthesia staff	Allied health personnel
Providers in Canadian hospitals	Attending anesthesiologists not targeted by onboarding
Providers in international hospitals	
Staff with less than one year of practice

Statistical analysis

Survey responses were summarized using descriptive statistics. Categorical variables are reported as counts and percentages. For 5-point Likert items, responses were treated as ordinal and summarized using the median and interquartile range (IQR), with full category distributions also reported. No inferential testing was performed, given the quality-improvement context and sample size. Analyses were conducted using spreadsheet-based descriptive calculations.

Proposed implementation plan

The proposed next step is the phased implementation of a standardized thoracic ERAS template bundle with defined governance. The bundle would be developed from established evidence-based thoracic ERAS guidelines and consensus recommendations, including published international thoracic surgery, perioperative oncology, and enhanced recovery frameworks [2,12-14]. Content would be owned by the thoracic anesthesia group, developed with multidisciplinary input, and maintained through version control and scheduled review. Delivery would prioritize EHR integration (e.g., a linked protocol and/or procedure-specific order set for eligible cases) with a printed point-of-care backup in thoracic operating areas.

Post-implementation evaluation would assess uptake, adherence to key elements, and selected process outcomes, with iterative refinement through Plan-Do-Study-Act (PDSA) cycles.

Ethical considerations

Participation in this project was entirely voluntary, and participants were free to withdraw at any point before submitting their responses. All responses were collected anonymously and treated as strictly confidential. No anticipated risks to participation were identified. The data collected will be used solely for quality improvement purposes and may be shared in a de-identified form for educational or research dissemination. Importantly, no patients were involved in the design or conduct of this study.

## Results

Respondent characteristics and clinical exposure

A total of 25 responses were received. Respondent characteristics are summarized in Table 2.

**Table 2:** Respondent characteristics and clinical exposure (N=25)

Characteristic	n (%)
Role	
Staff anesthesiologist (≥1 year practice)	20 (80)
Fellow	3 (12)
Resident	2 (8)
Administrative/academic role	
Yes	20 (80)
No	5 (20)
Years of anesthesia experience	
≥10 years	16 (64)
5–10 years	7 (28)
3–5 years	1 (4)
1–3 years	1 (4)
Age range	
30–39 years	10 (40)
40–49 years	10 (40)
≥50 years	5 (20)
Medical school location	
Canada	17 (68)
Other	8 (32)
Anesthesia training/certification location	
Canada	17 (68)
Other	8 (32)
Current practice country	
Canada	24 (96)
Other	1 (4)
Practiced anesthesia in countries other than where trained/currently work	11 (44)
Prior ERAS exposure in clinical care	
Yes, frequently	20 (80)
Yes, occasionally	3 (12)
No, not at all	2 (8)
Thoracic/lung isolation case exposure per year	
None	2 (8)
1–10 (occasional exposure)	11 (44)
11–25 (moderate exposure)	4 (16)
26–50 (frequent exposure)	1 (4)
>50 (regular thoracic practice)	7 (28)

Most respondents were staff anesthesiologists (20/25, 80%), and 20/25 (80%) reported holding an administrative and/or academic role. Most respondents reported ≥10 years of anesthesia experience (16/25, 64%), and the most common age ranges were 30-39 years (10/25, 40%) and 40-49 years (10/25, 40%). Most completed medical school in Canada (17/25, 68%) and completed anesthesia training/certification in Canada (17/25, 68%).

Exposure to thoracic anesthesia varied: 11/25 (44%) reported 1-10 thoracic/lung isolation cases per year, 7/25 (28%) reported >50 cases per year, 4/25 (16%) reported 11-25 cases per year, 1/25 (4%) reported 26-50 cases per year, and 2/25 (8%) reported no exposure. Most respondents reported prior exposure to ERAS in clinical care (20/25 (80%) frequently; 3/25 (12%) occasionally).

Perceptions of a standardized thoracic ERAS protocol

Most respondents indicated that access to a standardized thoracic ERAS protocol before surgery would be beneficial. Nineteen of 25 (76%) reported it would increase confidence in running a thoracic case at least “somewhat” (median 3, IQR 3-4). Most respondents also felt a standardized thoracic ERAS protocol would support integration into new hospital environments (22/25, 88% “somewhat/very much”) (median 3, IQR 3-4), would allow greater focus on patient optimization rather than learning local standards of care (23/25, 92% “somewhat/very much”) (median 4, IQR 3-4), and would reassure thoracic surgeons that consistent, evidence-based anesthesia care is being delivered even when working with new or rotating practitioners (22/25, 88% “somewhat/very much”) (median 3, IQR 3-4).

Concerns and barriers

**Figure 1:**
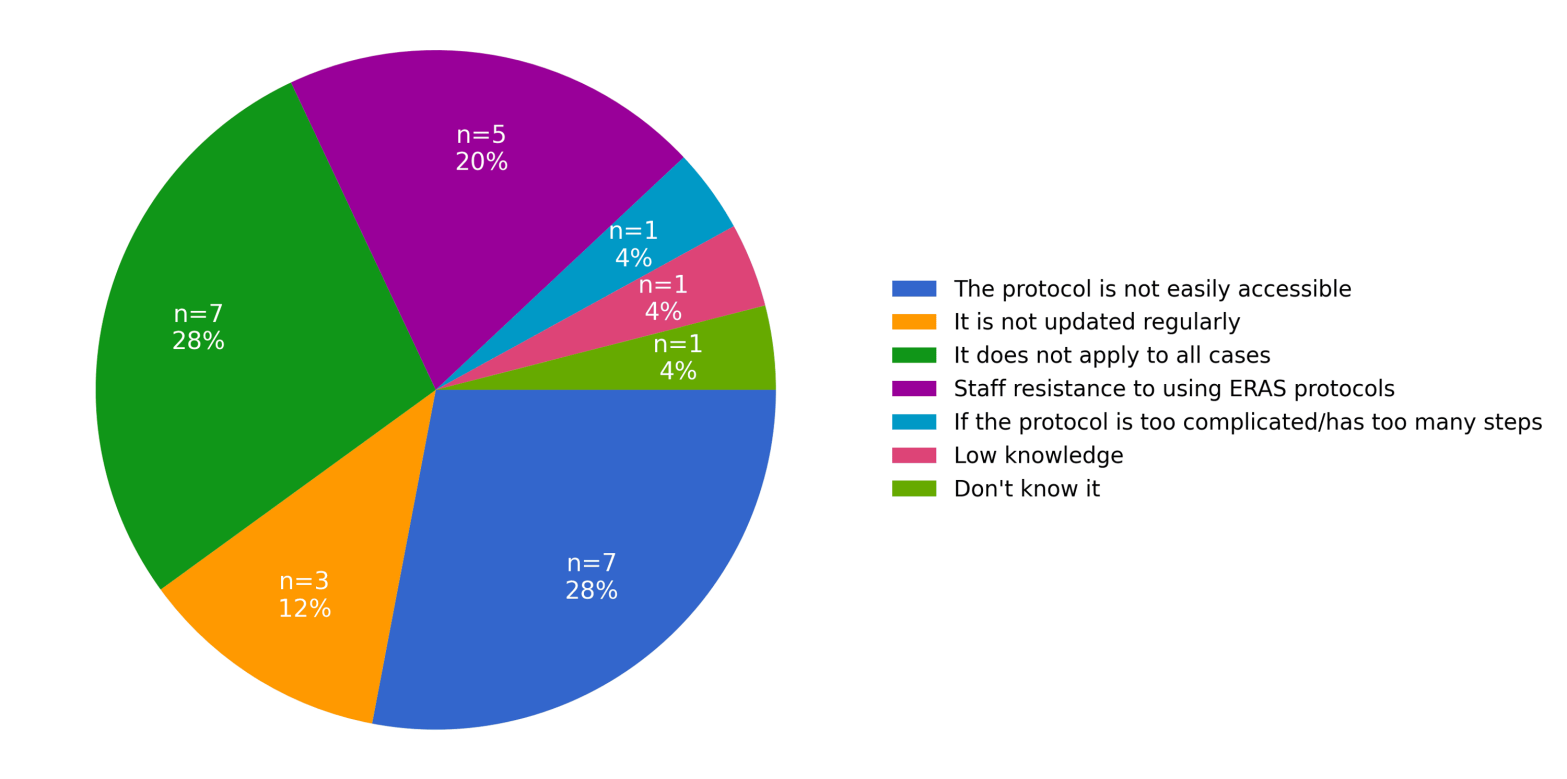
Main perceived barrier to the use of a standardized thoracic ERAS protocol (N=25) Single-choice responses to Question 20, identifying the primary barrier to protocol use (e.g., accessibility, applicability, staff resistance/buy-in, updating) ERAS: Enhanced Recovery After Surgery

Respondents reported several concerns regarding implementation (Figure 1).

Concern about reduced flexibility was mixed: 7/25 (28%) felt protocols could limit tailoring of care at least sometimes, 12/25 (48%) felt this would not be a concern, and 6/25 (24%) were unsure (median 2, IQR 1-3). In contrast, concern that a thoracic ERAS protocol could become outdated without regular review was common (21/25, 84% “yes, sometimes/definitely”) (median 3, IQR 3-4). Concern about over-reliance on protocols in place of clinical judgment was moderate (10/25, 40% “yes, sometimes”; 0/25 “yes, definitely”; 7/25 (28%) unsure) (median 2, IQR 1-3). Administrative burden was less frequently endorsed (4/25, 16% “yes, sometimes/definitely”), with most respondents reporting no or minimal concern (14/25, 56% “no, not at all/not really”) (median 1, IQR 0-2).

When asked to identify the single main barrier to using a standardized thoracic ERAS protocol, the most common selections were lack of easy accessibility (7/25, 28%) and limited applicability to all cases (7/25, 28%), followed by staff resistance/buy-in (5/25, 20%) and lack of regular updating (3/25, 12%). Smaller proportions selected low knowledge (1/25, 4%), not knowing the protocol (1/25, 4%), or protocol complexity (1/25, 4%).

Preferred access and learning modality

**Figure 2:**
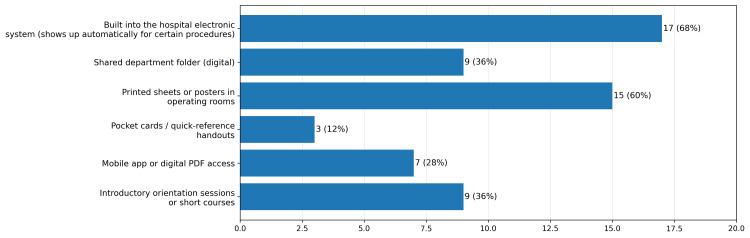
Preferred methods to access or learn about a thoracic ERAS protocol (N=25). Multi-select responses to Question 21 showing preferred dissemination modalities (EHR integration, printed materials, shared folder, orientation sessions, mobile/PDF access, pocket cards). Percentages may sum to >100% due to multiple selections. ERAS: Enhanced Recovery After Surgery; EHR: electronic health record

The preferred method was integration into the hospital electronic system/EHR (17/25, 68%), followed by printed sheets or posters in clinical areas (15/25, 60%). Other commonly selected options included a shared departmental digital folder (9/25, 36%) and introductory orientation sessions/short courses (9/25, 36%). Mobile app/digital PDF access was selected by 7/25 (28%), while pocket cards/quick-reference handouts were selected by 3/25 (12%) (Figure 2).

## Discussion

In this quality improvement needs assessment, thoracic anesthesia practitioners perceived that access to a standardized thoracic ERAS protocol prior to surgery could improve confidence in managing thoracic cases and support onboarding and integration when transitioning into new clinical environments. Respondents also emphasized that standardized guidance could help shift cognitive effort away from learning local norms and toward patient optimization and higher-value clinical decision-making. In addition, participants perceived that standardized protocols may reassure thoracic surgeons that anesthesia care remains consistent and evidence-based even when cases are managed by new or rotating practitioners.

These perceptions are consistent with literature describing the cognitive, logistical, and cultural challenges clinicians face when entering new practice settings, where variability in institutional protocols and expectations can contribute to uncertainty and inconsistent application of evidence-based practice [1]. This challenge is particularly relevant in thoracic anesthesia, where lung isolation, airway management, and perioperative planning for major lung resections require specialized knowledge and coordinated workflows. In perioperative care, adherence to structured pathways, such as ERAS protocols and institutional clinical guidelines, has been associated with improved outcomes and more efficient resource utilization, including after lung resection surgery [2,3]. However, as noted in prior work, onboarding processes often emphasize logistics while providing limited support for navigating professional expectations, local workflows, and team integration, which may increase safety risk and contribute to burnout [4-6].

Variation in anesthesia orientation practices may further compound these challenges. Residents transitioning into anesthesiology have described preoperative planning and case discussion as unfamiliar tasks characterized by inconsistent supervisory expectations and a lack of standardized formats, supporting the need for scaffolded learning and clearer communication frameworks [1]. Nationally, anesthesiology residency programs continue to report substantial variability in orientation content, timing, and delivery, with limited evidence linking specific approaches to objective performance outcomes [7,8]. Evidence from other specialties supports the value of more structured onboarding: for example, surgical onboarding interventions have improved perceived preparedness, situational awareness of local resources, and knowledge of key contacts [4]. Broader consolidation of practices into larger hospital systems further reinforces the importance of structured onboarding and mentorship to maintain consistent quality across diverse institutional contexts [5].

Respondents’ preference for integrating thoracic ERAS guidance into the electronic health record and other point-of-care formats aligns with emerging evidence that technology-enabled onboarding supports rapid access to institutional information. Prior work suggests that structured EHR training and onboarding interventions can improve confidence and promote adoption of best practices, while mobile and digital tools can strengthen team connectedness and reduce variability in day-to-day practice [9-11]. These findings support the feasibility of delivering standardized thoracic ERAS guidance through workflow-integrated methods, which may be more effective than passive dissemination alone.

Beyond an ERAS protocol, respondents’ comments support a broader “thoracic onboarding kit” concept that includes concise, thoracic-specific templates and cognitive supports. This may include category-level tools, such as a lung isolation checklist, a One-Lung Ventilation (OLV) troubleshooting algorithm (including stepwise responses to hypoxemia), emergency cognitive aids for high-stakes scenarios (e.g., hypoxemia, bleeding, airway loss with a double-lumen tube (DLT) in situ), and pathway summaries for lung resection and esophagectomy care. Published examples, such as the Canadian Thoracic Taskforce consensus cognitive aids for thoracic anesthesia emergencies, emphasize that cognitive aids are structured supports rather than rigid rulebooks; their effectiveness depends on appropriate training, easy access, and integration into local culture and routines [15]. These principles are directly relevant to implementing a standardized thoracic ERAS template that supports clinical judgment and team performance.

Implementation considerations identified in this assessment provide practical guidance for next steps. While views were mixed regarding whether standardized protocols might limit flexibility for individual patients, there was broader agreement that protocols require regular review to prevent outdated guidance. Some respondents also noted the risk of over-reliance at the expense of clinical judgment. Barriers to adoption centered on accessibility, applicability across diverse thoracic cases, and staff engagement or buy-in. These concerns suggest that successful implementation will require clear governance (content ownership and version control), scheduled updates, and explicit framing of protocols as decision support, with defined situations in which deviation is appropriate.

These needs-assessment findings constitute Phase 1 (Plan-Do-Study-Act (PDSA) Cycle 1) and provide the foundation for a planned post-implementation PDSA Cycle 2 evaluation of feasibility, uptake, and adherence. Future work should include a post-intervention evaluation to assess feasibility and adoption (e.g., protocol access and use), adherence to thoracic ERAS elements, and downstream effects on integration efficiency and selected clinical or operational outcomes. Such an evaluation would clarify real-world impact, identify unintended consequences, and inform the sustainability of thoracic anesthesia-specific onboarding templates in routine practice.

Interpretation of these findings should consider several limitations. The survey represents a needs assessment with a modest number of responses, largely from one health system, which may limit transferability and may reflect response bias toward clinicians interested in standardization. The respondent profile was weighted toward experienced staff anesthesiologists rather than learners. Because outcomes were perception-based and collected pre-implementation, the study does not evaluate objective changes in adherence, performance, or patient outcomes. Finally, exposure variables were self-reported, and the instrument was not a fully validated measure of constructs such as cognitive load or integration.

## Conclusions

This needs assessment of thoracic anesthesia practitioners, predominantly experienced staff anesthesiologists with variable thoracic case exposure, found strong perceived value in having a standardized thoracic ERAS protocol available before surgery. Respondents reported that such a tool would support confidence, facilitate faster integration into new practice environments, and enable greater focus on patient optimization rather than learning local standards of care. Key implementation considerations included preserving clinical flexibility and ensuring protocols are regularly reviewed to remain current, with electronic health record integration identified as the preferred access method. Overall, these findings support the feasibility and acceptability of standardized thoracic ERAS templates as an onboarding and practice-support intervention and justify a post-implementation evaluation using objective measures of uptake, adherence, and selected process or clinical outcomes.

## Appendices

Standardized templates for thoracic anesthesia practitioners: a quality improvement needs assessment to support onboarding and protocol adherence

You are invited to participate in a quality improvement project examining the usefulness of distributing standardized clinical templates (e.g., ERAS protocols, hospital guidelines, perioperative pathways) to support thoracic anesthesia practitioners during onboarding and clinical practice. This project aims to assess whether such tools enhance confidence and facilitate faster integration for residents, fellows, and staff while managing anesthesia for thoracic surgery cases.

Electronic Consent

By proceeding with this survey, you confirm that:

 Your participation is voluntary.

- Your responses will remain anonymous and confidential.

- You may withdraw at any time before submission by closing the survey window.

- There are no anticipated risks to participation.

- The data collected will be used for quality improvement and may be shared in de-identified form for educational or research purposes.

By clicking “I agree”, you consent to participate in this survey.

( ) I agree to participate

( ) I do not agree to participate

Sociodemographic Questions

1- Current role

· Anesthesia Resident (specify PGY level)

o PGY1

o PGY2

o PGY3

o PGY4

o PGY5

· Anesthesia Fellow

· Staff anesthesiologist (<1 year of practice)

· Staff anesthesiologist (≥1 year of practice)

2- Do you currently hold an administrative or academic role in your department?

· Yes

· No

3- Years of anesthesia experience (post-medical school)

· <1-3 year

· 3-5 years

· 5-10 years

· ≥10 years

4- Age range 

· <30 year

· 30-39 year

· 40-49 year

· 50+ year

5- Medical School

· Canada

· United States

· United Kingdom / Ireland

· Europe

· Middle East

· North Africa

· South Asia (e.g., India, Pakistan, Bangladesh, Sri Lanka, etc.)

· East / Southeast Asia (e.g., China, Japan, Philippines, etc.)

· Australia / New Zealand

· Other (please specify)

6- Where did you complete your official anesthesia training and certification (e.g., residency, master’s, PhD, diploma)?

· Canada

· United States

· United Kingdom / Ireland

· Europe

· Middle East

· North Africa

· South Asia (e.g., India, Pakistan, Bangladesh, Sri Lanka, etc.)

· East / Southeast Asia (e.g., China, Japan, Philippines, etc.)

· Australia / New Zealand

· Other (please specify)

7- Country of current practice

· Canada

· United States

· United Kingdom / Ireland

· Europe

· Middle East

· North Africa

· South Asia (e.g., India, Pakistan, Bangladesh, Sri Lanka, etc.)

· East / Southeast Asia (e.g., China, Japan, Philippines, etc.)

· Australia / New Zealand

· Other (please specify)

8- Have you practiced anesthesia in countries other than where you trained or currently work?

· Yes

· No

9- Your current hospital setting (select all that apply)

· Academic teaching hospital

· Community hospital

· Tertiary/quaternary referral center

· Other (please specify)

10- Have you ever provided anesthesia care for a case that was managed using an Enhanced Recovery After Surgery (ERAS) protocol?

· Yes, frequently

· Yes, occasionally

· No, not at all

11- What best describes your level of exposure to thoracic surgeries or cases requiring lung isolation techniques (e.g., one-lung ventilation, double-lumen tube, bronchial blockers)?

· None (no exposure)

· 1-10 cases per year (occasional exposure)

· 11-25 cases per year (moderate exposure)

· 26-50 cases per year (frequent exposure)

· More than 50 cases per year (regular thoracic practice)

Perceptions of Standardized Templates

12- If a standardized Enhanced Recovery After Thoracic Surgery (ERAS) protocol was provided before surgery, how much would this increase your confidence in running the case?

· Very much

· Somewhat

· Neutral

· Very little

· Not at all

13- How much would a standardized thoracic ERAS protocol help you integrate faster into new hospital environments during rotations or new appointments?

· Very much

· Somewhat

· Neutral

· Very little

· Not at all

14- How much would having a standardized thoracic ERAS protocol allow you to focus more on patient optimization and clinical questions, rather than spending time learning the “local standard of care”?

· Very much

· Somewhat

· Neutral

· Very little

· Not at all

15- How much would a standardized ERAS protocol reassure thoracic surgeons that consistent, evidence-based anesthesia care is provided, even when working with new or rotating practitioners?

· Very much

· Somewhat

· Neutral

· Very little

· Not at all

Barriers and Concerns

16- Do you think using an ERAS protocol during thoracic surgery could limit flexibility in tailoring care for individual patients?

· Very much

· Somewhat

· Neutral

· Very little

· Not at all

17- Would you be concerned that a thoracic ERAS protocol might become outdated if not regularly reviewed?

· Very much

· Somewhat

· Neutral

· Very little

· Not at all

18- Do you think a thoracic ERAS protocol could make people rely too much on them instead of using their own clinical judgment?

· Very much

· Somewhat

· Neutral

· Very little

· Not at all

19- Do you think introducing a thoracic ERAS protocol would add extra paperwork or administrative burden?

· Very much

· Somewhat

· Neutral

· Very little

· Not at all

20- What do you see as the main barrier to using a standardized thoracic ERAS protocol? (single choice)

· The protocol is not easily accessible

· It takes too much time to use during cases

· It is not updated regularly

· It does not apply to all cases

· Staff resistance to using ERAS protocols

· Other (please specify)

21- How would you prefer to access or learn about a thoracic ERAS protocol? (Select all that apply)

· Built into the hospital electronic system (shows up automatically for certain procedures)

· Shared department folder (digital)

· Printed sheets or posters in operating rooms

· Pocket cards / quick-reference handouts

· Mobile app or digital PDF access

· Introductory orientation sessions or short courses

· Other (please specify)
